# Prions and Prion-Like Pathogens in Neurodegenerative Disorders

**DOI:** 10.3390/pathogens3010149

**Published:** 2014-02-18

**Authors:** Caterina Peggion, Maria Catia Sorgato, Alessandro Bertoli

**Affiliations:** Department of Biomedical Sciences, University of Padova, Viale G. Colombo 3, Padova 35131, Italy; E-Mails: caterina.peggion@unipd.it (C.P.); catia.sorgato@unipd.it (M.C.S.)

**Keywords:** prion, prion-like, PrP, neurodegeneration, amyloid-β, tau, α-synuclein, Alzheimer’s disease, Parkinson’s disease

## Abstract

Prions are unique elements in biology, being able to transmit biological information from one organism to another in the absence of nucleic acids. They have been identified as self-replicating proteinaceous agents responsible for the onset of rare and fatal neurodegenerative disorders—known as transmissible spongiform encephalopathies, or prion diseases—which affect humans and other animal species. More recently, it has been proposed that other proteins associated with common neurodegenerative disorders, such as Alzheimer’s and Parkinson’s disease, can self-replicate like prions, thus sustaining the spread of neurotoxic entities throughout the nervous system. Here, we review findings that have contributed to expand the prion concept, and discuss if the involved toxic species can be considered *bona fide* prions, including the capacity to infect other organisms, or whether these pathogenic aggregates share with prions only the capability to self-replicate.

## 1. Prion Diseases, Prions and Prion Strains

Prion diseases are devastating neurodegenerative disorders affecting humans and different animal species. The agent of these fatal illnesses has been sought at least since the early twentieth century, in the context of the scrapie epidemics in sheep and goats. The culpable pathogen had long been suspected of having unorthodox properties [1,2], but it attracted worldwide attention after provoking the “mad cow” disease (or bovine spongiform encephalopathy, BSE) in the mid-1980s [3], and the finding that BSE-contaminated food could transmit disease to humans [4]. These events fueled momentum to conclusively define the agent’s nature and the mechanism of replication [5]. In addition to scrapie, prion diseases—also denominated transmissible spongiform encephalopathies (TSE) to highlight the extensive vacuolization and loss of neurons in diseased brains—include chronic wasting disease (CWD) in cervids, and BSE in cattle, kuru, Creutzfeldt-Jacob disease (CJD) (with its variant form (vCJD) caused by BSE prions), and fatal familial insomnia in humans.

After the isolation and characterization of the TSE agent, the original hypothesis by Griffith [6] was formally proven by Stanley Prusiner (reviewed in Prusiner, 1998) [7], that the prion (“proteinaceous infectious particle”) consists mainly of an aggregated, aberrant, and self-replicating conformer (PrP^Sc^) of the cellular prion protein (PrP^C^) [8,9], a normal protein widely expressed in mammalian tissues, and localized to the cell surface. As in other neurodegenerative disorders, TSE are mostly idiopathic in origin and typically late-onset, while the genetic forms (accounting for approximately 10% of total TSE, and with point or insertional mutations in the PRNP gene) have a slightly earlier age at onset and a slower course [10]. Iatrogenic cases have been reported, due to contaminated neurosurgical instruments [11,12], brain extracts (e.g., pituitary growth hormone) [13], corneal transplantation [14], dura mater graft [15], and, in most recent years, transfusion of vCJD prion-infected blood [16].

The casual, genetically- or infectivity-linked misfolding of the essentially α-helical PrP^C^ [17] entails a β-sheet enrichment of the aberrant PrP^Sc^ isoform [18], whose sticky surface domains favor attraction and conformational conversion of other PrP^C^ molecules, leading to the formation of soluble oligomers of increasing length. In this chain reaction of elongation, PrP^Sc^ functions as a template that imposes its aberrant structure on native PrP^C^ molecules [7,19]. The change in conformation drastically modifies PrP^C^ biological properties, e.g., PrP^Sc^ shows increased resistance to proteolysis (e.g., by proteinase K) [8], suggesting that the acquired compacted structure could contribute to the resistance of prions to external harsh environments (e.g., the digestive tract). Oligomers, and larger aggregates, can fragment, amplifying in this way the prion seeding capacity [20], and may ultimately deposit outside neurons as insoluble amyloid fibrils, characterized by ordered β-sheet repeats perpendicularly oriented to the fiber axis.

Accounting for cognitive and motor syndromes typical of many TSE-affected individuals, there is plentiful evidence that prions can invade large areas of the central nervous system (CNS) from the site at which PrP^Sc^ originates [21], either spontaneously or following exposure to exogenous prions. Thus, by diffusing within the extracellular space [22], prions are able to propagate from neuron to neuron, but can also exploit complex extraneural routes (e.g., lymphoid organs) [23] to reach the CNS, as when prions are orally [24], or parenterally [16], introduced.

All prion diseases have in common PrP^C^ misfolding, yet they are characterized by variable clinical presentations, affecting brain area and biochemical features, among and within species. Increasing evidence indicates that these properties, ranging from incubation periods and histopathological profiles to biochemical properties of PrP^Sc^, all depend on the specific conformation of PrP^Sc^ present in a prion [25]. Prions can thus exist as strains encoded by the conformation of PrP^Sc^ and not by modified genetic information as in the case of bacterial and viral strains. It was also found that the “barrier” of recipients to the attack of prions from another species may diminish upon serial transmission [26], in accord with the increasing variations of the biochemical and pathogenetic features of the original prion [27]. This, and newer findings, have thus suggested that a prion strain behaves as a quasi-species, consisting of mixed PrP^Sc^ conformers [28,29], and that prevalence of a “substrain” depends on the constraints imposed by the environment [29,30]. The substrain selection resembles that of viral and bacterial mutants, but in this case the mutant is the PrP^Sc^ conformation that best adapts to a new environment. Following these results, the emergent new concept is that prions evolve in Darwinian terms despite the absence of nucleic acids, whereby the most advantaged conformer is selectively amplified [29].

## 2. Extending the Prion Paradigm

The now popular prion paradigm—namely, (i) recruitment of benign monomers by aggregates of misfolded proteins that impose their anomalous structure on native polypeptides; (ii) growth and fragmentation of oligomers; (iii) spreading of the pathology—has been extended to other neurodegenerative disorders. Indeed, misfolded β-sheet-enriched proteins associated with neuronal deterioration—e.g., amyloid-β (Aβ) fragments, tau, α-synuclein (α-syn), polyglutamine (polyQ) expansion-containing proteins, and superoxide dismutase-1, implicated in Alzheimer’s disease (AD), tauopathies, Parkinson’s disease (PD), and Huntington’s disease (HD)/ataxia, respectively—and forming extra- or intra-cellular amyloids with cross β-pleated sheet conformation were reported to self-replicate, and are, therefore, increasingly denominated prions [31,32] or prion-like proteins. However, the former definition should be used with caution, because the capacity of a misfolded protein to colonize the CNS (or other organs) should not be confused with the capacity to transmit the disease from an affected individual to a healthy recipient, *i.e.*, to obey Koch’s main principle—that an agent responsible for a given disease is defined infectious if it causes the same disease when inoculated into a susceptible host. To date, adherence to Koch’s postulates has been definitively proven for only (true) prions, including maintenance of the self-replicating capacity over multiple serial passages from one animal to another, and the *in vivo* contagious properties, which allow passing the disease within animals (scrapie, CWD), or within humans (iatrogenic CJD, kuru), or from animals to humans (vCJD).

It is important to remember that amyloids are also the signature of systemic amyloidoses, a group of fatal diseases in which organs other than the CNS accumulate ordered aggregates of proteins, including transthyretin, immunoglobulin light chains and islet amyloid polypeptide [33]. However, no evidence for their transmission between individuals is available, except for Amyloid A seeds [34]. Another example of amyloids, yet with an opposite biological significance, are functional amyloids that diverse organisms (bacteria, yeast and humans) produce to accomplish functions that are key to the organism physiology [35,36]. For example, most mammalian peptide hormones are apparently stored as amyloids in secretory granules [37].

## 3. AD, Tau- and Synuclein-opathies

AD is the most common cause of dementia among the elderly. The classical pathological markers of AD are senile plaques and neurofibrillary tangles (NFT). Senile plaques are extracellular aggregates of Aβ 1-40/1-42 peptides, deriving from the sequential proteolysis (by β- and γ-secretases) of the transmembrane amyloid precursor protein (APP) of unknown function [38]. AD is also characterized by the intracellular accumulation of NFT, which are composed of hyper-phosphorylated tau protein (for a recent review see Wang *et al*., 2013) [39] that, in its native form, plays a significant role in stabilizing neural microtubules [40,41]. Both Aβ and tau oligomers have been implicated in disease pathogenesis (reviewed in Ashe and Zahs, 2010) [42].

Recognition of the spread of Aβ deposits (from the entorhinal cortex to all hippocampal subregions and cortical areas) as an AD sign, and that AD progression is sustained by the self-propagation of Aβ oligomers, was based on both clinical observations of AD pathology (in humans and animal models), and on intracerebral inoculation of AD brain extracts (in nonhuman primates and human APP-expressing transgenic mice) resulting in accelerated Aβ deposition [43,44,45,46,47,48,49]. Brain Aβ spread was also observed using inocula of synthetic Aβ peptides [50], or by injecting AD brain extracts in the peritoneal cavity [51]—although a longer incubation period was needed. Recently, more than 20 Aβ peptide variants have been reported in the cerebrospinal fluid of AD patients [52]. One of these, lacking the first two amino acids of Aβ 1-40 and 1-42 fragments and with the *N*-terminal glutamate enzymatically modified to pyroglutamate, had the capacity to recruit canonical Aβ 1-42 into toxic oligomers, which then propagated with a prion-like mechanism [53]. Together with other types of evidence, this finding strongly indicates that, like prions, Aβ peptides may fold in different manners, thus giving rise to “strains” with specific pathologic aspects [47,54].

If, in principle, no obstacle should prevent Aβ oligomers from diffusing within the extracellular space to anatomically-close regions, the location of NFT inside cells implies that NFT need to pass two plasma membranes to invade other neurons [55,56]. Parenthetically, if present alone NFT are a hallmark of tauopathies, a group of neurodegenerative disorders (frontotemporal lobar degeneration and chronic traumatic encephalopathy, among others) with specific brain lesions that call, once again, for the existence of (tau) “strains” [57]. Thus, in both AD and tauopathies [43,58], a similar mechanism could allow the diffusion of NFT in neighboring neurons, possibly at the level of synapses [59]. Several putative mechanisms allowing release, transport and uptake could bypass the need of tau seeds to cross membranes, ranging from microvesicles and exosomes to tunneling nanotubules, or other unconventional routes including membrane pores formed by misfolded aggregates themselves [60,61,62,63,64,65,66]. A final answer to this question is, however, not yet available.

The issue of how intracellular seeds propagate is common to other disease-related inclusion bodies, such as Lewy bodies present in the neuronal somata and processes of patients affected by synucleinopathies, a group of neurodegenerative disorders that include PD, the second most prevalent neurodegenerative disorder. Polymerized misfolded β-sheet-rich α-syn is the most abundant component of Lewy bodies. This intrinsically unfolded protein (but see Bartels *et al*., 2011) [67] is enriched in presynaptic nerve terminals [68,69] where it performs a still unclear function, although some data suggest an implication in the recycling of synaptic vesicles and neurotransmitter release [70,71]. Each synucleinopathy is invariably associated with α-syn deposits, yet it displays a typical clinical syndrome. Possibly, this is due to the capacity of α-syn to generate distinct fibrillar conformers [72], which *in vivo* could then determine the primary affected brain area, the specific clinical outcome and disease progression.

As in AD, the progressive spread of PD to different areas of the brain [73] has suggested that diffusion and replication of α-syn oligomers occur in a prion-like manner [74]. Several *in vitro* and *in vivo* data support the capacity of α-syn oligomers to be released from, and be taken up by neighbouring, neurons, also when pre-formed fibrils are added [75,76,77,78,79,80,81,82,83,84]. Yet, the strongest evidence is the observation that Lewy bodies developed in embryonic neuronal cells after more than a decade from their transplantation in the striatum of PD patients [85,86]. This indicates that α-syn deposits, escaped from the patient’s neurons, were internalized by healthy neurons in which they acted as seeds to recruit endogenous α-syn.

All of this evidence, and other examples of similar prion-like behaviors, e.g., the nuclear deposits of poly-Q proteins present in HD and spinocerebellar ataxia [87], indicate that a large number of neurodegeneration-linked misfolded proteins can colonize the CNS from the site at which protein misfolding and aggregation first occur [88]. In spite of the still elusive mechanism for the delivery of intracellular aggregates, they also suggest that the self-assembly and propagation properties are probably intrinsic to oligomeric species of β-enriched misfolded proteins, irrespective of the type of monomeric precursor.

## 4. Mechanism of Toxicity

Another common issue in neurodegenerative disorders, in which a decade-long asymptomatic phase generally precedes the onset of clinical symptoms, is the identification of the species responsible for neuronal death, and of the mechanism underlying the phenotypic manifestation of the disease.

For a long period of time, it was generally accepted that misfolded protein deposits—be they plaques, tangles or inclusion bodies—had intrinsic toxic properties [89] and that their toxicity correlated with disease progression. Other observations, however, now undermine the validity of this concept. While the strongest support comes from some genetic TSE forms that do not present PrP^Sc^ deposits [90], other examples are: the improvement of cognitive functions of a mouse model with reduced soluble tau, yet with intact NFT [91]; the failure of immune therapies to cure AD dementia although clearing Aβ amyloids from the brain [92]; a mutant form of Aβ, associated with AD dementia, which favored oligomerization over fibril formation [93]. The poor correlation between the presence of amyloids and cognitive impairment, together with the high thermodynamic stability typical of large fibrillar deposits (but deviations have been suggested, see Tycko and Wickner, 2013 [94]), has thus led to the proposal that small and soluble assemblies of disease-related misfolded proteins are the primary species causing synaptic dysfunction and neuronal demise [95,96,97,98,99,100].

Concurrently, several lines of evidence support the hypothesis that the mechanisms of propagation and toxicity are distinct. A clear example pertains to prion diseases in which PrP^Sc^ would be responsible for the exponential phase of prion replication coinciding with increased prion infectivity. However, in the subsequent phase characterized by saturated prion replication, PrP^Sc^ oligomers would catalyze formation of another conformer, denoted PrP^L^, which, once it has reached a local threshold concentration, would become toxic to neurons and initiate clinical manifestations [28,101]. Thus, two distinct PrP species, corresponding to the two (silent and overt clinical) phases of prion diseases, apparently exist: PrP^Sc^ with self-replicating and infectious capacity, and PrP^L^ with neurotoxic properties. After all, that misfolded prion proteins have different properties was first proven in a transgenic mouse expressing a TSE-related PrP^C^ mutant, which ultimately died but had no infectivity associated with its brain extracts [102]. In spite of some uncertainties, all these results place TSE in a more advanced stage for understanding the disease origin than most of the other neurodegenerative disorders.

Whichever the species inducing neuronal damage in the different diseases, alternative to the “gain-of-toxicity” view is the concept that recruitment of native proteins by the seeding mechanism, or the altered physiology *per se* of the pathologic isoform, could deprive cells of key functions ultimately resulting in neuronal impairment and death. This “loss-of-function” mechanism was undeniably proven in the case of spinocerebellar ataxia type 1, caused by expansion of a glutamine-encoding repeat in ataxin 1, in which the amount of nuclear deposits of mutant ataxin-1 was shown to inversely correlate with the severity of the disease [103]. Subsequently, polyQ/ataxin-1 was found to be unable to form as many complexes with a transcriptional regulator as could wild-type ataxin-1 [104]. This suggests that incapacity of a protein to perform the required task leads to neurodegeneration. Unfortunately, the incompletely disclosed role of PrP^C^, α-syn or APP/Aβ peptides has precluded thus far full understanding of whether a loss-of-function phenotype underlies the association of these proteins with neurodegeneration, nor have gene knockout experiments provided conclusive evidence in favor of the loss-of-function hypothesis. Indeed, mice lacking PrP^C^, α-syn, or APP/Aβ displayed no evident phenotype, or only subtle abnormalities that did not resemble the respectivediseases [70,105,106,107,108].

Interestingly, data are now available that could enable the formulation of a unifying principle for diverse disorders in which both concepts (gain-of-toxicity and loss-of-function) contribute to neurotoxicity. Once again, a major input came from examining *in vivo* the role of PrP^C^ in prion diseases. These studies demonstrated that the presence of PrP^C^—physiologically bound to the neuronal surface—was essential not only for forming prions, but also for initiating disease [109,110,111]. Subsequent *in vitro* studies finally proved that indeed the *N*-terminus of native PrP^C^ is able to interact with PrP^Sc^, and that PrP^C^ conveys prion’s deadly signal to neurons only if properly inserted into detergent-insoluble domains of the plasma membrane [112]. Noteworthy, PrP^C^ needs to be properly inserted into these domains also for carrying out its cell-protective functions [112,113].

Several studies have extended the principle that PrP^C^ may act as a (co)-receptor for misfolded entities to cause neuronal demise: by binding Aβ oligomers with high affinity, and mediating memory impairment of transgenic AD mice [114,115]; by transducing the toxic message of a variety of β-sheet rich proteins, of different length and origin [112,116]. One can therefore conceive that attachment of misfolded proteins to PrP^C^ alters the physiologic conformation and membrane insertion of PrP^C^, and, hence, its native function. Accordingly, it was observed that, in the presence of PrP^C^, Aβ oligomers alter the activity of the Src family tyrosine kinase Fyn [100,117], a protein that is presumably downhill of the PrP^C^-regulated signaling pathway [118], and is implicated in Aβ-induced synaptic deficit and cognitive dysfunction [119,120,121]. Along this line, also glutamate receptors have been implicated. On the one hand, it was reported that ionotropic NMDA receptors, which are known to mediate Aβ oligomers’cytotoxicity and to be down-regulated by PrP^C^ [122,123], are necessary for PrP^C^ transduction of Aβ toxic message [116]. On the other hand, by interacting physically with PrP^C^, the metabotropic glutamate receptor mGluR5 was shown to act as a connecting partner allowing Aβ-PrP^C^ complex at the cell surface to activate cytosolic Fyn and the downhill processes ultimately leading to neuronal dysfunction [124].

## 5. Conclusions

Several *in vitro* and *in vivo* data have been accumulated lending support to the possibility that the above-mentioned terms of the prion concept are applicable to a number of neurodegeneration-linked oligomers. This is important, because adherence to the prion paradigm may open multiple perspectives for development of early diagnosis, prevention and therapeutic strategies for these, as yet incurable, disorders.

Concurrently, a possible new means of elucidating the mechanism provoking neuronal impairment and demise has been provided by studies demonstrating the capacity of PrP^C^ to bind misfolded β-sheet-enriched proteins of different origin, and to transmit their deadly message inside neurons. This issue is not yet unanimously accepted, e.g., the validity of the PrP^C^-dependent effects of Aβ oligomers on cognitive functions [125]. Nonetheless, the molecular and cellular inputs originating from this proposition will likely provide unprecedented information on the poorly understood causes of neuronal injury and death, including consequences deriving from impairment of the normal function of PrP^C^.
